# Parental academic involvement and academic buoyancy among junior high school students: cross-sectional indirect associations involving peer relations and academic emotions

**DOI:** 10.3389/fpsyg.2026.1832439

**Published:** 2026-08-07

**Authors:** Miaomiao Wang, Xunbao Yin, Yuhan Zhao, Shuai Teng, Xiang Ji

**Affiliations:** School of Education, Weifang University, Weifang, China

**Keywords:** academic buoyancy, academic emotions, junior high school students, parental academic involvement, peer relations

## Abstract

**Objective:**

This study examined the association between parental academic involvement and academic buoyancy among junior high school students from families meeting predefined parental-education and household-income criteria, and tested whether peer relations and academic emotions were involved in cross-sectional statistical indirect-association patterns between these variables.

**Methods:**

Using a cross-sectional survey design, we collected online data from 867 Chinese junior high school students through the Wenjuanxing platform. Measures assessed parental academic involvement, peer relations, academic emotions, and academic buoyancy. After controlling for gender, grade, and only-child status, we conducted Pearson correlation analyses and cross-sectional parallel statistical indirect-association analyses. Bootstrap procedures with 5,000 resamples were used to estimate statistical indirect associations and their 95% confidence intervals.

**Results:**

Parental academic involvement was positively associated with positive academic emotions and academic buoyancy, and negatively associated with peer fear and inferiority and negative academic emotions. Parallel statistical indirect-association analyses showed significant total indirect-association patterns in both models (maternal academic involvement: effect = 0.0380, 95% CI [0.0290, 0.0485]; paternal academic involvement: effect = 0.0283, 95% CI [0.0196, 0.0379]). In both models, significant specific statistical indirect associations were observed in the patterns involving peer fear and inferiority, negative academic emotions, and positive academic emotions, with the largest statistical indirect association involving positive academic emotions. By contrast, the indirect association involving peer acceptance was not significant in either model.

**Conclusion:**

In this cross-sectional sample, academic buoyancy was concurrently associated with parental academic involvement, peer relational experiences, and academic emotions, particularly positive academic emotions.

## Introduction

Within school settings, adolescents' academic adjustment is shaped not only by a small number of acute adverse events, but also by recurrent and cumulative forms of everyday academic stress, such as fluctuations in grades, homework load, exam evaluation, and classroom comparison and feedback (Martin and Marsh, 2008). Although such stressors do not typically constitute major adversity in the classical sense, their high frequency, prolonged duration, and deep embedding in school life can exert a sustained influence on students' learning engagement, emotional states, and quality of adjustment (Steare et al., 2023). Whether students can maintain functioning and re-engage in the face of these everyday academic challenges is therefore central to understanding their academic development. In this context, Martin and Marsh (2008) and Martin and Marsh (2009) defined academic buoyancy as students' capacity to deal successfully with the routine academic setbacks and pressures of school life. At its core, the construct captures how ordinary students maintain functioning, persist, and re-engage under everyday learning-related stress.

Junior high school is a particularly relevant period for examining academic buoyancy. On the one hand, students entering this stage typically face more frequent academic evaluation, heavier learning demands, and stronger examination-oriented pressure. On the other hand, self-awareness develops rapidly during this period, while peer relations, academic emotions, and performance feedback become increasingly salient to academic adjustment (Evans et al., 2018). As a result, junior high school students in highly competitive educational environments are more likely to experience sustained and normalized academic pressure (Goldstein et al., 2015). A recent systematic review further noted that, although research on academic resilience has identified a wide range of protective factors, substantial heterogeneity remains in the definition of “adversity,” the operationalization of outcomes, and research design. Against this backdrop, academic buoyancy offers a more precise framework for understanding how ordinary students adapt to the routine pressures of school life (Ye et al., 2024).

Adolescents' adaptation to these everyday academic pressures, however, is not shaped by stress alone; it is profoundly embedded in their developmental contexts. As the earliest and most stable context of socialization, the family continues to influence adolescents' emotional development, behavioral regulation, and school adjustment even after the onset of puberty (Steinberg, 2000). Prior work has shown that family context not only shapes emotional development in children and adolescents, but also plays a central role in the formation of emotion regulation capacities (Morris et al., 2007). Parents' attention to, participation in, and investment in their children's learning relate not only to direct learning support, but also to adolescents' understanding of academic demands, perceptions of academic stress, and ways of coping. Meta-analytic evidence indicates that parental involvement in schooling is generally associated with students' academic performance, learning engagement, and socioemotional adjustment, although this association is not unidirectional or uniform, and different forms of involvement may correspond to different developmental outcomes (Barger et al., 2019). In addition, parental educational involvement is negatively associated overall with adolescent depressive symptoms, although this association is moderated by age, grade level, and the source of involvement information (Liu et al., 2024). These findings suggest that parental academic involvement is not merely a surface-level behavior of “providing help,” but a family-context variable associated with adolescents' academic adjustment and with cognitive, emotional, and coping-related correlates of school experience.

This family-resource perspective is especially relevant for junior high school students from families with relatively advantaged educational and economic resources, because parental academic involvement may take different forms depending on family resource background. In the present study, this subgroup was defined operationally rather than as a formal social class category. Families in this subgroup were expected to have both educational capital, reflected in at least one parent having completed college education, and household-level economic capacity, reflected in an annual household income of RMB 200,000 or higher. These resources may provide relatively stable conditions for supporting children's schooling, such as familiarity with academic expectations, access to educational information, and the capacity to invest in learning-related activities. However, relative resource advantage does not necessarily translate into secure educational advantages, particularly in highly competitive educational contexts. Under such conditions, parents' attention to children's schooling may simultaneously reflect support, expectation management, and concerns about educational uncertainty (Luo and Zhu, 2024; Wang et al., 2024). Parental academic involvement is therefore a family educational variable that is especially relevant to examining academic buoyancy in this specific family-resource context.

By the time students enter junior high school, family influences do not remain confined to the home; rather, they increasingly manifest through relational experiences in everyday school life. As peer interaction becomes more central, whether adolescents feel accepted, supported, or threatened by peer evaluation can shape their sense of school belonging, relational security, and behavioral engagement (Somerville, 2013). Research has shown that peer acceptance is associated with more active academic engagement, and that school belonging is consistently linked to students' motivation, socioemotional functioning, behavioral engagement, and academic outcomes (Korpershoek et al., 2020; Lan, 2023). A recent systematic review further emphasized that the development of school belonging is inherently ecological and reflects the combined relevance of individual characteristics as well as family, peer, teacher, and school factors (Štremfel et al., 2024). Recent evidence also suggests that peer acceptance and rejection during secondary school are significantly associated with subsequent educational outcomes (Plenty and la Roi, 2024). At the same time, adolescents' peer relations do not develop independently of family context. Prior work has shown that parent–child relationship quality, as well as parenting characteristics such as parental acceptance, responsiveness, and involvement, shape adolescents' integration into the peer world (Deković and Meeus, 1997). A systematic review further indicated that parental attachment and parent–child relationship quality are consistently associated with the quality of adolescents' peer relationships (Delgado et al., 2022). Peer relations can thus be understood as an important proximal social context that is relevant to both family-related experiences and school adjustment.

Corresponding to peer relations as a social condition, adolescents' adaptation to academic demands is also reflected in their emotional responses to learning tasks. Control-value theory posits that social environments shape students' academic emotions by influencing their perceptions of control and value (Pekrun, 2024). Positive academic emotions are typically associated with higher learning engagement, more flexible cognitive processing, and more effective self-regulation, whereas negative academic emotions are more likely to accompany resource depletion and sustained impairment in adaptation (Pekrun, 2024). Recent longitudinal research has likewise shown that adolescents' social-emotional skills are associated with higher school engagement and lower school burnout (Huttunen et al., 2024). In this process, parental academic involvement remains highly relevant. Previous studies suggest that parental involvement may be related to adolescents' academic emotions through its links with academic value and academic self-efficacy (Dong et al., 2020). At the same time, supportive parent–child interactions are associated with more positive academic coping, whereas negative parent–child interactions are more likely to predict avoidant and disengaged coping (Zimmer-Gembeck et al., 2023). These findings suggest that parental academic involvement relates not only to learning behavior itself, but also to adolescents' subjective appraisals of learning tasks and emotional experiences, thereby bearing on how they adapt to routine academic stress. Academic emotions therefore represent an important psychological correlate to consider when examining associations among family context, school relational experiences, and academic buoyancy.

Within this framework, the present study focused on junior high school students from families with relatively advantaged educational and economic resources and examined whether parental academic involvement was associated with academic buoyancy. We further examined whether peer relations and academic emotions showed distinct cross-sectional indirect-association patterns when considered simultaneously. Although theory allows for sequential or reciprocal relations between peer experiences and academic emotions, the present study specified these variables in parallel because the same-wave data could not identify their temporal ordering. The proposed model was therefore intended to compare their conditional statistical associations within a common framework, rather than to represent a developmental sequence or test causal mediation.

## Methods

### Participants and procedure

We conducted an online survey through the Wenjuanxing platform among adolescents from families meeting predefined parental-education and household-income criteria. This family-resource background was operationally identified using two a priori screening criteria: (1) at least one parent had completed college education or above, and (2) annual household income was RMB 200,000 or higher. The parental education criterion was used to indicate the presence of family educational capital, because parents with college education may be more familiar with academic expectations, school progression, and learning-related support. The household income criterion was used to indicate relatively greater household-level economic capacity to support children's schooling. The two criteria were used jointly because parental education and household income capture different but complementary aspects of family resources. Importantly, these thresholds were not intended to correspond to an official national socioeconomic classification, nor were they intended to provide a comprehensive measure of socioeconomic status. Rather, they were used as pragmatic sampling criteria to identify families with both educational and economic resources relevant to parental academic involvement.

**Figure 1:**
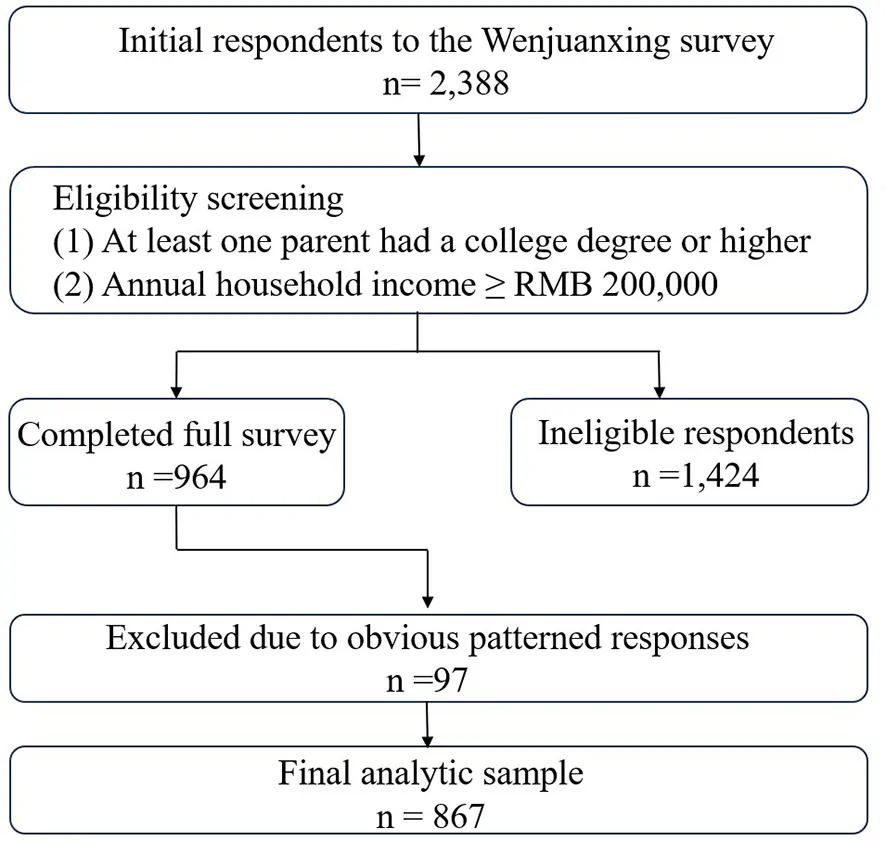
Flowchart of participant screening and inclusion.

Eligible participants completed a demographic questionnaire and the formal study measures. Questionnaires were excluded if they showed clear patterned responding, such as selecting the same response option across a large number of consecutive items, highly repetitive response sequences, or logically inconsistent responses to reverse-scored items. As shown in Figure 1, 2,388 respondents entered the Wenjuanxing survey, 964 met the eligibility criteria and completed the full survey, 97 were excluded due to clear patterned responding, and the final analytic sample comprised 867 participants.

### Ethics statement

This study involving human participants was reviewed and approved by the Ethics Committee of Weifang University. The study was conducted in accordance with local legislation and institutional requirements. Before participation, all participants and their legal guardians were provided with information about the study purpose, anonymity, voluntary participation, and the right to withdraw at any time. Electronic informed consent was obtained from the participants' legal guardians, and assent was obtained from the participants before data collection.

### Measures

#### Parental academic involvement

We assessed parental academic involvement using the *Junior High School Students' Parental Involvement Scale* developed by Song (2010). The scale includes two subscales, one for paternal involvement and one for maternal involvement. Each subscale contains 21 items and covers three dimensions: emotional involvement, intellectual involvement, and behavioral management involvement. All items are rated on a 5-point scale, with higher scores indicating higher levels of parental involvement. In the present study, Cronbach's alpha was 0.896 for the paternal involvement subscale, 0.906 for the maternal involvement subscale, and 0.942 for the total scale.

#### Academic emotions

We measured academic emotions using the Adolescent Academic Emotions Questionnaire developed by Dong and Yu (2007). The scale was developed based on two dimensions of academic emotions, valence and arousal, and includes four subscales: positive high-arousal emotions, positive low-arousal emotions, negative high-arousal emotions, and negative low-arousal emotions. All items are rated on a 5-point scale, with higher scores indicating stronger corresponding academic emotional experiences. Because the present study focused on broad emotional valence, the two positive subscales were combined into a positive academic emotions composite, and the two negative subscales were combined into a negative academic emotions composite. In the present study, Cronbach's alpha was 0.886 for positive academic emotions, 0.926 for negative academic emotions, and 0.967 for the full scale.

#### Peer relations

We assessed peer relations using the revised *Peer Relations Scale* developed by Zou (1998). The scale includes a peer acceptance subscale (Items 1–20) and a peer fear and inferiority subscale (Items 21–30), for a total of 30 items. Items are rated on a 4-point scale. In the peer acceptance subscale, all items except 1, 3, 4, 7, 11, and 17 are reverse-scored. Higher scores on the peer acceptance subscale indicate higher levels of acceptance and support in peer interactions, whereas higher scores on the peer fear and inferiority subscale indicate stronger experiences of fear and inferiority in peer contexts. In the present study, Cronbach's alpha was 0.784 for peer acceptance, 0.901 for peer fear and inferiority, and 0.923 for the total scale.

#### Academic buoyancy

We measured academic buoyancy using the *Academic Buoyancy Scale* originally developed by Martin and Marsh and revised in Chinese by Sun (2009). The scale contains four items rated on a 5-point scale. Higher total scores indicate a stronger capacity to recover, persist, and re-engage under everyday academic pressure. In the present study, Cronbach's alpha for this scale was 0.816.

### Data analysis

We used SPSS 26.0 to conduct reliability analyses, the common method bias test, descriptive statistics, and Pearson correlation analyses. We then used PROCESS 4.2 Model 4 to estimate cross-sectional parallel statistical indirect-association models, entering paternal academic involvement and maternal academic involvement separately as independent variables, academic buoyancy as the dependent variable, and peer acceptance, peer fear and inferiority, positive academic emotions, and negative academic emotions as candidate variables in the indirect-association model. Gender, grade, and only-child status were included as basic demographic covariates in the primary models. To examine whether the findings were sensitive to covariate selection, we further conducted sensitivity analyses using expanded covariate models by adding family residence, family structure, and the corresponding parent's education and occupation to the primary covariates. Statistical indirect associations were estimated using 5,000 bootstrap resamples and their 95% confidence intervals; these estimates were considered statistically significant when the confidence interval did not include zero.

## Results

### Common method bias

Because all core variables were assessed via student self-report, we examined common method bias using Harman's single-factor test. An unrotated exploratory factor analysis of all measured items yielded 32 factors with eigenvalues greater than 1, and the first unrotated factor accounted for 14.07% of the total variance, which was below the conventional 40% threshold (Tang and Wen, 2020). This result suggests that no single dominant factor accounted for most of the item covariance.

### Descriptive statistics and bivariate correlations

Descriptive statistics and bivariate correlations for parental academic involvement, peer relations, academic emotions, and academic buoyancy are presented in Table 1.

**Table 1:** Descriptive statistics and correlations among study variables.

Variable	*M*	*SD*	1	2	3	4	5	6	7
1. Paternal academic involvement	83.35	22.26	1						
2. Maternal academic involvement	87.54	21.18	0.815^**^	1					
3. Academic buoyancy	14.15	3.60	0.252^**^	0.263^**^	1				
4. Peer fear and inferiority	20.21	7.05	−0.142^**^	−0.120^**^	−0.241^**^	1			
5. Peer acceptance	58.12	7.54	0.080^*^	0.051	0.151^**^	−0.583^**^	1		
6. Negative academic emotions	109.36	26.88	−0.144^**^	−0.119^**^	−0.264^**^	0.487^**^	−0.371^**^	1	
7. Positive academic emotions	104.31	17.33	0.284^**^	0.329^**^	0.493^**^	−0.083^*^	0.055	−0.156^**^	1

*N* = 867; *M*, mean; SD, standard deviation.

^*^p < 0.05; ^**^p < 0.01.

Both paternal and maternal involvement were positively associated with academic buoyancy and positive academic emotions, and negatively associated with peer fear and inferiority and negative academic emotions. Academic buoyancy was negatively associated with peer fear and inferiority and negative academic emotions, and positively associated with peer acceptance and positive academic emotions. These correlations provided descriptive background for the subsequent cross-sectional indirect-association analyses.

### Cross-sectional parallel indirect-association analyses

Cross-sectional parallel indirect-association analyses were conducted using PROCESS 4.2 Model 4, with paternal and maternal academic involvement entered separately as independent variables, academic buoyancy as the dependent variable, and peer fear and inferiority, peer acceptance, positive academic emotions, and negative academic emotions as candidate variables in the statistical indirect-association model. Gender, grade, and only-child status were controlled in all equations. Statistical indirect associations were estimated using 5,000 bootstrap resamples and were considered significant when the 95% bootstrap confidence interval did not include zero. Given the cross-sectional design, these estimates should be interpreted as statistical indirect associations rather than evidence of temporal or causal mediation.

The total statistical indirect associations were significant in both the maternal involvement model (effect = 0.0380, Boot SE = 0.0050, 95% Boot CI [0.0290, 0.0485]) and the paternal involvement model (effect = 0.0283, Boot SE = 0.0047, 95% Boot CI [0.0196, 0.0379]). In both models, significant statistical indirect associations were observed in the patterns involving peer fear and inferiority, negative academic emotions, and positive academic emotions, whereas the indirect association involving peer acceptance was not significant. Overall, paternal and maternal academic involvement showed similar cross-sectional indirect-association patterns: higher parental academic involvement was associated with lower peer fear and inferiority, lower negative academic emotions, and higher positive academic emotions, and these variables were concurrently associated with higher academic buoyancy.

**Figure 2:**
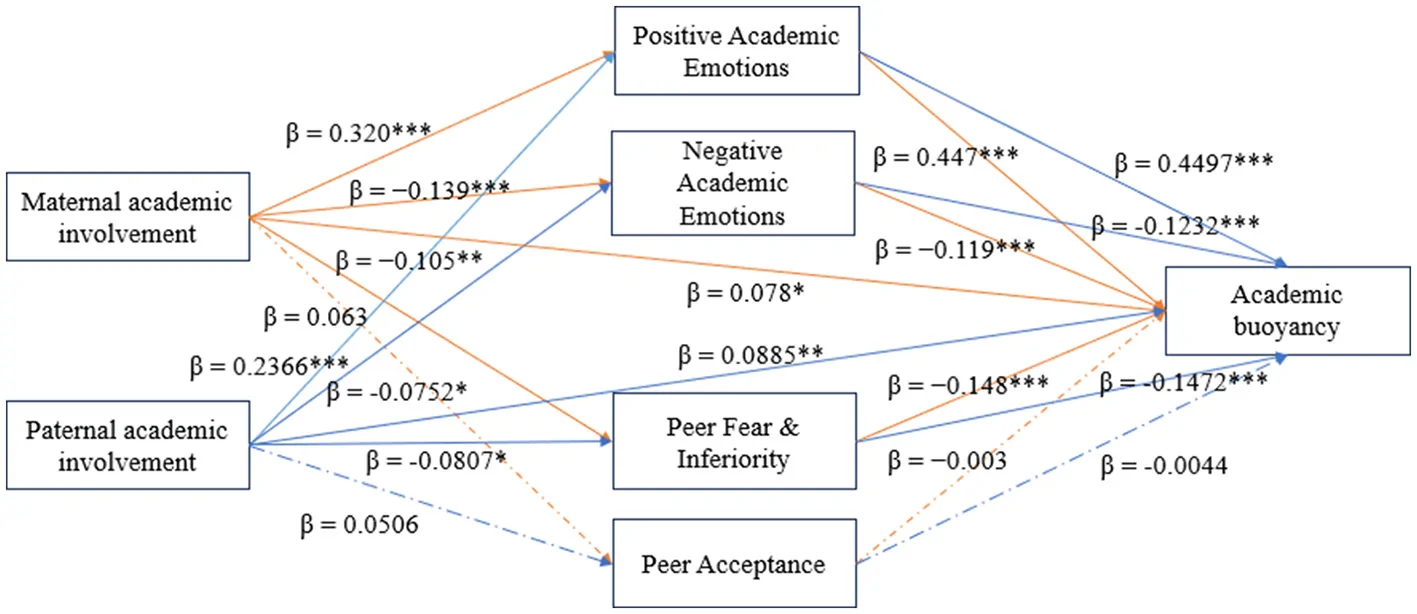
Cross-sectional indirect-association model of paternal and maternal academic involvement and academic buoyancy. Note. Standardized path coefficients (β) are shown. Blue lines represent the paternal involvement model, and orange lines represent the maternal involvement model. Solid lines indicate statistically significant coefficients, whereas dashed lines indicate non-significant coefficients. *p<*0.05*. **p<*0.01*. ***p<*0.001*.

The maternal and paternal involvement models explained 30.40% and 30.60% of the variance in academic buoyancy, respectively. Specific statistical indirect associations are presented in Table 2, and the cross-sectional indirect-association model is shown in Figure 2.

**Table 2:** Specific statistical indirect associations between maternal and paternal academic involvement and academic buoyancy.

Statistical indirect-association pattern	*B*_ind_	SE	95% CI	β_ind_
Maternal academic involvement → Peer fear and inferiority → Academic buoyancy	0.0034	0.0016	[0.0009, 0.0069]	0.0155
Maternal academic involvement → Peer acceptance → Academic buoyancy	0.0000	0.0007	[−0.0018, 0.0013]	−0.0002
Maternal academic involvement → Negative academic emotions → Academic buoyancy	0.0036	0.0015	[0.0011, 0.0070]	0.0165
Maternal academic involvement → Positive academic emotions → Academic buoyancy	0.0311	0.0048	[0.0222, 0.0413]	0.1431
Paternal academic involvement → Peer fear and inferiority → Academic buoyancy	0.0026	0.0014	[0.0004, 0.0059]	0.0119
Paternal academic involvement → Peer acceptance → Academic buoyancy	0.0000	0.0006	[−0.0015, 0.0011]	−0.0002
Paternal academic involvement → Negative academic emotions → Academic buoyancy	0.0021	0.0012	[0.0001, 0.0048]	0.0093
Paternal academic involvement → Positive academic emotions → Academic buoyancy	0.0236	0.0045	[0.0154, 0.0330]	0.1064

*B*_ind_, unstandardized indirect-association estimate; β_ind_, completely standardized indirect-association estimate, reported as an effect-size indicator.

Bootstrap estimates were based on 5,000 resamples.

Confidence intervals that do not include zero indicate statistically significant indirect associations.

### Sensitivity analyses

Sensitivity analyses using expanded covariate models produced substantively similar results to the primary models. After additionally controlling for family residence, family structure, and the corresponding parent's education and occupation, the total statistical indirect associations remained significant in both the maternal involvement model (effect = 0.0366, 95% CI [0.0279, 0.0467]) and the paternal involvement model (effect = 0.0273, 95% CI [0.0186, 0.0367]). The pattern of specific indirect associations was unchanged: in both models, the indirect associations involving negative academic emotions, positive academic emotions, and peer fear and inferiority remained significant, whereas the indirect association involving peer acceptance remained non-significant.

## Discussion

### Main findings and contributions

This study examined the association between parental academic involvement and academic buoyancy among junior high school students from families meeting predefined educational and household-income criteria, and further tested cross-sectional indirect-association patterns involving peer relations and academic emotions. Both paternal and maternal involvement were positively associated with academic buoyancy. After controlling for gender, grade, and only-child status, both forms of parental involvement showed significant indirect-association patterns involving peer fear and inferiority, negative academic emotions, and positive academic emotions, with the largest estimate involving positive academic emotions. By contrast, the indirect association involving peer acceptance was not significant.

The present study contributes to the literature in three specific ways. First, it focuses on students from families with relatively advantaged educational and economic resources, a group for whom parental academic involvement may reflect not only support but also expectation management under educational competition. Second, it examined paternal and maternal involvement in parallel rather than treating parental involvement as a single undifferentiated construct. Third, it compared multiple concurrent indirect-association patterns involving peer relations and academic emotions, thereby showing which correlates were more prominent in the present data.

These findings extend the contextualized understanding of academic buoyancy. Academic buoyancy refers to students' capacity to manage routine academic setbacks and everyday school pressures rather than recovery from rare major adversity. The present results suggest that academic buoyancy is associated not only with individual adjustment, but also with family-, peer-, and emotion-related correlates. Thus, the theoretical value of the study lies not simply in reaffirming that parental involvement is related to academic buoyancy, but in situating this association within a broader pattern of peer and emotional experiences.

### Parental academic involvement and academic buoyancy

Both paternal and maternal involvement were positively associated with academic buoyancy. This suggests that even during junior high school, when autonomy and peer influence become increasingly salient, the family remains a relevant context for adolescents' everyday academic adjustment. In the family-resource context examined here, parental academic involvement may reflect support, expectation management, and risk management under educational uncertainty. Therefore, parental involvement should not be treated as a neutral family variable or interpreted as a simple “more is better” process.

A balanced interpretation is that higher parental academic involvement was generally associated with higher academic buoyancy in this sample, while the meaning of this association may depend on the quality and subjective experience of involvement. The measure used in this study captured overall parental input in emotional, intellectual, and behavioral management domains, but could not distinguish whether adolescents experienced such involvement primarily as support, monitoring, or pressure.

### Indirect associations involving peer relations and academic emotions

The main indirect-association pattern was that parental academic involvement showed statistical indirect-association patterns involving peer fear and inferiority, negative academic emotions, and positive academic emotions. Peer fear and inferiority showed a significant indirect-association pattern, whereas peer acceptance did not. Positive academic emotions showed the strongest estimated indirect association. These findings highlight the relevance of peer-related threat experiences and academic emotions, while the non-significant result for peer acceptance should not be taken as evidence that peer acceptance is unimportant.

Peer acceptance and peer fear and inferiority may represent related but distinct dimensions of peer experience. Peer acceptance reflects positive affiliation, inclusion, and interpersonal support, whereas peer fear and inferiority reflects perceived social threat and vulnerability to negative evaluation. This distinction may help explain why peer acceptance was positively correlated with academic buoyancy but did not show a unique indirect association after the other candidate variables were included. It is also possible that peer acceptance operates through constructs not included in the present model, such as school belonging, classroom participation, or academic self-concept.

Measurement factors should also be considered. The reliability of the peer acceptance subscale was acceptable but lower than that of the other measures, with Cronbach's alpha of 0.784 whereas most other scales exceeded 0.90. This relatively lower reliability may have introduced greater measurement error and attenuated the observed association involving peer acceptance. Therefore, the non-significant indirect association should be interpreted as a finding from the present measurement and model specification rather than as evidence that peer acceptance has no relevance to academic buoyancy.

The findings regarding academic emotions point to the most prominent statistical association pattern in the study. According to control-value theory, academic emotions are related to students' appraisals of control and value and to their attention, self-regulation, and persistence. In the present data, parental academic involvement showed indirect-association patterns involving both negative and positive academic emotions, but the estimate involving positive academic emotions was larger. This suggests that academic buoyancy under everyday academic pressure may be linked not only to lower anxiety, boredom, or frustration, but also to interest, hope, engagement, and positive task orientation.

The distinction between statistical significance and practical significance is important. Although several indirect associations were statistically significant, the standardized indirect-association estimates involving peer fear and inferiority and negative academic emotions were very small. These effects should not be overinterpreted as strong or practically decisive pathways. The more defensible practical implication is that academic buoyancy appears to be embedded in a broader pattern of family involvement, peer experiences, and academic emotions, with positive academic emotions showing the most prominent association in the present data.

Both the total indirect association and the specific indirect association involving positive academic emotions were somewhat larger in the maternal involvement model than in the paternal involvement model. This difference should be interpreted with restraint because the study did not directly assess the functional qualities or subjective meanings of paternal and maternal involvement. The present findings support the conclusion that both paternal and maternal involvement were positively associated with academic buoyancy, while possible differences in the strength and structure of these associations require further investigation.

### Limitations, practical implications, and future directions

Several limitations should be acknowledged. First, the study used a cross-sectional design, and all paths should be understood as theoretically informed associations rather than causal relations (Maxwell and Cole, 2007). Reverse or reciprocal associations are plausible; for example, academically buoyant students may perceive parental involvement more positively, report more positive academic emotions, and experience less peer-related threat. For this reason, we did not interpret the ratio of indirect to total associations as a causal proportion mediated. Future research should adopt longitudinal, cross-lagged, experience-sampling, or experimental designs to test temporal ordering more rigorously.

Second, all core variables were assessed through student self-report at a single time point. Although Harman's single-factor test did not indicate a dominant general factor, this procedure cannot rule out common method variance. Future studies should incorporate multiple informants and methods, such as parent reports of academic involvement, teacher ratings of academic adjustment, peer nominations of social relations, and classroom behavioral or learning-process indicators.

Third, the sample was restricted to junior high school students from families meeting predefined parental-education and household-income criteria. This sampling strategy helped focus the study on a family educational context that is particularly relevant under contemporary educational competition in China, but it also limits generalizability. Future studies should examine whether the present association patterns are stable across different socioeconomic groups and should use more comprehensive socioeconomic indicators.

Fourth, we combined academic emotions into positive and negative indicators. Although this decision improved model clarity, it may have obscured differences between emotions with different levels of arousal. Future research should differentiate more finely among types of academic emotions to determine whether they are differentially associated with academic buoyancy.

Fifth, the parallel indirect-association framework was an analytical simplification rather than a complete developmental model. Peer relations and academic emotions may be sequentially or reciprocally related, but testing such patterns requires temporally separated measurements. Future longitudinal work should compare parallel, serial, and reciprocal models more directly.

Despite these limitations, the findings carry practical implications. At the family level, parental academic involvement should be considered not only in terms of the amount of supervision or performance expectations, but also in terms of how adolescents perceive its stability, supportiveness, and emotional meaning. At the school level, students who report persistent peer fear, inferiority, or withdrawal may require attention, although the small effect estimates suggest that these variables should be viewed as part of a broader risk pattern rather than as isolated intervention targets. Positive academic emotions may be especially useful to monitor and cultivate when supporting students' everyday academic adjustment.

## Conclusion

Overall, parental academic involvement was positively associated with academic buoyancy among junior high school students. In the statistical indirect-association models, this association was characterized by lower peer fear and inferiority, lower negative academic emotions, and especially higher positive academic emotions. These cross-sectional findings indicate that adolescents' academic buoyancy under everyday academic pressure is concurrently related to parental academic involvement, peer relational experiences, and academic emotions.

## Data Availability

The raw data supporting the conclusions of this article will be made available by the authors, without undue reservation.
